# Mood Disorders Induced by Maternal Overnutrition: The Role of the Gut-Brain Axis on the Development of Depression and Anxiety

**DOI:** 10.3389/fcell.2022.795384

**Published:** 2022-01-26

**Authors:** Jeferson Jantsch, Isadora D’Ávila Tassinari, Márcia Giovenardi, Victorio Bambini-Junior, Renata Padilha Guedes, Luciano Stürmer de Fraga

**Affiliations:** ^1^ Programa de Pós-Graduação em Biociências, Universidade Federal de Ciências da Saúde de Porto Alegre (UFCSPA), Porto Alegre, Brazil; ^2^ Programa de Pós-Graduação em Ciências Biológicas: Fisiologia, Universidade Federal do Rio Grande do Sul (UFRGS), Porto Alegre, Brazil; ^3^ Programa de Pós-Graduação em Ciências da Saúde, Universidade Federal de Ciências da Saúde de Porto Alegre (UFCSPA), Porto Alegre, Brazil; ^4^ School of Pharmacy and Biomedical Sciences, University of Central Lancashire (UCLan), Preston, United Kingdom; ^5^ Division of Biomedical and Life Sciences, Faculty of Health and Medicine, Lancaster University, Lancaster, United Kingdom

**Keywords:** maternal obesity, gut-brain axis, anxiety, depression, microbiota

## Abstract

Since the first evidence suggesting that maternal nutrition can impact the development of diseases in the offspring, much has been elucidated about its effects on the offspring’s nervous system. Animal studies demonstrated that maternal obesity can predispose the offspring to greater chances of metabolic and neurodevelopmental diseases. However, the mechanisms underlying these responses are not well established. In recent years, the role of the gut-brain axis in the development of anxiety and depression in people with obesity has emerged. Studies investigating changes in the maternal microbiota during pregnancy and also in the offspring demonstrate that conditions such as maternal obesity can modulate the microbiota, leading to long-term outcomes in the offspring. Considering that maternal obesity has also been linked to the development of psychiatric conditions (anxiety and depression), the gut-brain axis is a promising target to be further explored in these neuropsychiatric contexts. In the present study, we review the relationship between maternal obesity and anxious and depressive features, exploring the gut-brain axis as a potential mechanism underlying this relationship.

## Introduction

The Developmental Origins of Health and Disease (DOHaD) theory brought by Barker and Osmont (1986) states that prenatal environmental factors influence the offspring’s health in adulthood (Barker and Osmond, 1986). Both malnutrition and overnutrition are determinants of metabolic and neurological dysfunctions in the offspring, such as impairments in the brain reward circuitry, neuroinflammatory states, and behavioural disorders (Smith and Reyes, 2017).

Neurodevelopmental disorders are well documented in the offspring of obese mothers in humans and animal models (Edlow, 2017; Tong and Kalish, 2021). However, observational epidemiological studies have struggled to establish a direct cause-effect relationship between gestational obesity and neurological diseases in the progeny. Nevertheless, in preclinical studies, there is a substantial body of evidence showing a mechanistic link between maternal obesity and neurological dysfunctions in the offspring (Edlow, 2017). For example, long-term hippocampal insulin resistance in obese mice dams was followed by a decrease in the expression of neurogenesis markers and synaptic plasticity in the offspring (Schmitz et al., 2018). Moreover, the impact of maternal obesity is multigenerational, impairing synaptic function in the hippocampus until the third generation (Fusco et al., 2019).

Considering that obesity during pregnancy is increasing globally (Chen et al., 2018), as well as the prevalence of mood disorders such as depression and anxiety (WHO, 2017), it is important to unravel the mechanisms linking these conditions. Maternal obesity alters intestinal microbiota (Chu et al., 2016), which has emerged as a key regulator of metabolism and behaviour. Thus, understanding how the maternal intestinal microbiota influences fetal neurodevelopment is fundamental to comprehend the repercussions of maternal obesity on the mental health of the offspring (Kumpulainen et al., 2018).

This review will discuss the influence of maternal overnutrition on the development of depression and anxiety in the offspring. We will emphasize the role of the gut-brain axis in the mechanisms linking maternal obesity to the harmful neurodevelopment of the progeny.

## Depression

Intrauterine overnutrition could affect the fetal programming, increasing the risk of the newborn developing neuropsychiatric conditions such as depression (McDonald et al., 2015; Cárdenas-Tueme et al., 2020; Cattane et al., 2021).

Offspring of rodent mothers fed with a high-fat diet (HFD) during pregnancy and lactation shows increased depressive-like behaviour in adolescence and adulthood (Gawlinska et al., 2019), even if the offspring was fed a standard diet. Although there is some controversy in the literature [for review see (Ortiz-Valladares et al., 2021)], it reinforces that prenatal exposure to maternal HFD is a decisive factor that can lead to depressive symptoms later in life (Gawlinska et al., 2019). Transcriptional changes in the prefrontal cortex (Gawlińska et al., 2021), reduction in the number and morphology of dendrites and spines in the hippocampus, amygdala, and somatosensory cortex (Hatanaka et al., 2017), lower volume of brain areas such as the thalamus, hippocampus, nucleus accumbens, and hypothalamus, in addition to changes in synaptophysin and GFAP (glial fibrillary acidic protein) expression (Trujillo-Villarreal et al., 2021) may be part of the mechanisms underlying the behavioural changes seen in the adult offspring.

Maternal exposure to HFD, or a carbohydrate-rich diet, also affects the dopaminergic (DA) system by changing the amount of dopamine and its metabolite DOPAC (3,4-dihydroxyphenylacetic acid) in the nucleus accumbens and the expression of dopamine receptors D1/D2 in the ventral tegmental area (Naef et al., 2008; Naef et al., 2011; Sullivan et al., 2015; Paradis et al., 2017). Dysregulation of these circuits increases the risk of mood disorders and altered function of the mesolimbic DA system, as well as the dopamine receptor in limbic structures, has already been observed in different models of depression (Winter et al., 2007; Tye et al., 2013; Belujon and Grace, 2017).

Clinical studies have reported that elevated levels of proinflammatory cytokines in obese pregnant women can lead to placental dysfunction and are related to neurodevelopmental and neuropsychiatric disorders in the offspring [for review see (Howell and Powell, 2017)]. Evidence from humans and animals also links depressive-like behaviour with immune system disturbances or neuroinflammatory responses. Proinflammatory mediators such as C-reactive protein, interleukin (IL)-6, IL-1β, and tumor necrosis factor (TNF)-α affect the development of neural circuits that are critical for regulation of behaviour, providing evidence that inflammation may also be a potential factor leading to increased risk of mood disorders (Rivera et al., 2015; Rasmussen et al., 2019).

Furthermore, emotional changes in the progeny were associated with maternal nutritional status (Ortiz-Valladares et al., 2021) and high maternal body mass index with a decline in psychological development in the early stages of childhood (Bergmann et al., 2016; Aubuchon-Endsley et al., 2017). In summary, poor maternal nutrition during pregnancy negatively impacts neurodevelopmental outcomes in the first years of life and plays an important role in inducing mental disorders in the offspring.

## Anxiety

Maternal obesity has been associated with offspring’s anxiety independently of sex, species, and age (Menting et al., 2019). However, the mechanisms linking maternal obesity to the development of anxiety in the offspring remain a gap in the field. The available evidence will be briefly scrutinized here.

During adolescence, there is an increase in anxiety in the offspring of HFD-fed dams, shown in the light-dark transition task, but a decrease in anxiety in the open field and elevated plus-maze, which could be interpreted as an increased risky behaviour rather than decreased anxiety. Importantly, an increase in the expression of nuclear factor kappa B (NF-kB) and IL-6 genes, responsible for inflammatory responses, was found in the hippocampus of adolescent offspring of dams fed with a hypercaloric diet. But an increase in the expression of anti-inflammatory genes such as IkBα and mitogen-activated protein kinase MKP-1 was also seen, suggesting a disturbance in the expression of inflammatory mediators in the brain of these animals (Sasaki et al., 2014). On the other hand, when analyzing the offspring of HFD-fed dams during adult life, an increase in anxiety-like behaviours was observed in the open field, elevated plus-maze, and light-dark transition tasks. The maternal diet also changed the concentration of plasma corticosteroids after stress challenge and the expression of pro-inflammatory genes in the amygdala, such as NF-kB or IL-6, with a notable sex-specific influence (Sasaki et al., 2013). Although the effect of maternal diet on anxiety did not appear to be influenced by sex or age in the systematic review by Menting et al. (2019), it is clear that this claim is controversial and needs further investigation.

Most studies involving maternal consumption of obesogenic diets use the HFD model. However, the cafeteria diet (CAF) model provides animals with ultra-processed human food consumption and has translational potential (Matuszewska et al., 2021). Wright et al. showed that maternal consumption of a CAF impacts offspring behaviour, regardless of whether consumption occurs in the preconception period, during pregnancy, lactation, or all these periods (Wright et al., 2011). Interestingly, CAF during lactation caused a consistent anxiolytic effect in males as observed by the increase of entries into and more time spent on open arms in the elevated plus-maze and the reduced latency to enter the center in the open field test. It reinforces that the sex of the offspring is an important factor and should be taken into account when studying the effects of maternal obesity (Wright et al., 2011).

Otherwise, consuming CAF exclusively during pregnancy increased maternal behaviours such as licking pups, arched nursing, and nest building after 2- and 8-days following birth. Interestingly, even with increased maternal behaviour, the offspring demonstrated behavioural disturbances in adolescence such as increased anxiety-like manifestations during light-dark tests (Ribeiro et al., 2018).

Table 1 summarizes preclinical studies demonstrating maternal diet’s impact on depressive and anxiety-like behaviours in the progeny.

**TABLE 1:** Effects of maternal nutrition on depressive and anxious-like behaviour and reported mechanisms in preclinical studies.

Reference	Maternal diet	Offspring species and diet	Molecular and behavioural findings in the progeny
**Depression**			
Gawlinska et al. (2019)	HFD (60%) pregnancy and lactation	Wistar rats–SD	↑ Anhedonia and depressive-like behaviour in the FST
			↓ Irisin in serum and hippocampus of females on PND 28
			↓ Interleukin-1α in the hippocampus of females on PND28 and PND63
Gawlińska et al. (2021)	HFD (60%) pregnancy and lactation	Wistar rats–SD	↑ Anhedonia and depressive-like behaviour in the FST and SPT
			Transcriptomics changes in the prefrontal cortex
de la Garza et al. (2019)	CAF, HFD and HSD	Wistar rats–SD	↑ Anhedonia and depressive-like behaviour in the FST
			↑ TBK1 after HSD in the hippocampus
Trujillo-Villarreal et al. (2021)	CAF	Wistar rats–SD	↓ Motivation for natural rewards on OCT, SPT, and SFT
			↓ Frontomesocorticolimbic circuit volume
			↓ Synaptic terminals in the hippocampus and nucleus accumbens
			↑ GFAP in the hippocampus and hypothalamus
			↓ Number of hippocampal cells
			↓ Myelin in the dentate gyrus of hippocampus
			↑ GluR1 and GluR2 subunits of AMPA receptors
			↓ mGluR2 expression in the hippocampus
**Anxiety**			
Bruce-Keller et al. (2017)	HFD	C57BL/6 mice–SD	↑ Anxiety-like behaviour in the open field in males
			↑ Marble buying in males; Changes in microbial diversity in males and females
Matuszewska et al. (2021)	CAF	Wistar rats–SD	At PND25, CAF offspring
			↑ % of fat content; sex-specific differences in glucose levels
			↑ Serum IL-6, IL-10, and TNF-α in females
			Sex-specific differences in concentration of IL-6 and TNF-α, ↑ in CAF females
			↑ Serum IL-10 in males
Sasaki et al. (2013)	Perinatal HFD	Long Evans rats–SD	↓ Anxiety-like behaviour in adult animals
			Selective alteration in the GR expression and inflammatory genes in the hippocampus and amygdala
			↑ Corticosterone after stressful challenges in females
Sasaki et al. (2014)	Perinatal HFD	Long Evans rats–SD	↑ Center entries (females) and ↑ time spent in the center (males and females) in the OF
			↑ Open arm entries in the elevated plus maze (both sexes)
			↑ GR transcript in the hippocampus of the females
			↓ Hippocampal gene expression of NF-*κ*B, IL-6, IkBα, and MKP-1 in females
			↓ Gene expression of NF-*κ*B and IL-1Ra in the amygdala of females
Ribeiro et al. (2018)	CAF	Swiss mice–SD	↓ Latency for the first transition in the light-dark test
			↑ Number of transitions and time spent in the dark of light-dark test in males
			↓ Play behaviour
Wright et al. (2011)	Pre-gestational, gestational and lactational CAF diet	Wistar rats–SD	↓ Anxiety in males shown by the OF test

AMPA, α-amino-3-hydroxy-5-methyl-4-isoxazolepropionic acid; CAF, cafeteria diet; FST, forced swimming test; GFAP, glial fibrillary acidic protein; GluR, glutamate receptor; GR, glucocorticoid receptor; HFD, high-fat diet; HSD, high-sugar diet; IL, interleukin; IL-1Ra, interleukin-1 receptor antagonist; IkBα, I-kappa-B-alpha; MKP-1, mitogen-activated protein kinase 1; NF-*κ*B, nuclear factor kappa B; OCT, operant conditioning test; OF, open field; PND, postnatal day; SD, standard diet; SPT, sucrose preference test; SFT, suppressed feeding test; TKB1, TANK binding kinase-1; TNF-α, tumor necrosis factor-alpha.

## The Gut-Brain Axis During Fetal Development

Data presented so far reinforce a causative relationship between maternal diet and neurologic programming in offspring. However, the underlying mechanism involved in this phenomenon remains elusive. Despite that, the influence of maternal microbiota on the development of anxious behaviours in the offspring has emerged as a mechanism that is sufficient to disrupt behavioural function in murine offspring in a sex-specific manner (Bruce-Keller et al., 2017). It suggests that intestinal dysbiosis could link the effects of unhealthy modern diets to the increased prevalence of neurodevelopmental and childhood disorders.

In addition to fermenting non-digestible fibers from the diet, which increases energy extraction from food, the influence of the gut microbiota on the host’s physiology has been gaining notoriety from studies that demonstrate a bi-directional relationship with the brain, a pathway coined as the gut-brain axis (Martin et al., 2018). Although this communication is not completely clear, three main pathways are proposed: neural signals carried out by the vagus nerve; chemical signals synthesized by the microbiota, as short-chain fatty acids (SCFA) or neurotransmitters; and immunomodulation exerted by microbiota and its products (Morais et al., 2021).

The main phyla of bacteria that colonize the human gut are Firmicutes and Bacteroidetes (Eckburg et al., 2005), but microbiota composition is extremely dynamic and altered even in physiological contexts such as pregnancy. For example, in the first trimester, the maternal gut microbiota is more similar to that of men and non-pregnant women, while Proteobacteria and Actinobacteria are expanded during the third trimester (Koren et al., 2012). Conversely, analysis from 1479 pregnant women found no differences compared to age-matched non-pregnant women, suggesting individual heterogeneity as the most relevant element for changes in gut microbiota during pregnancy (Yang et al., 2020). Even though, in animal studies, mice that received faecal transplantation from women in the third trimester, but not in the first, develop weight gain and insulin resistance (Koren et al., 2012). Also, an increase in *Akkermansia, Clostridium, Bacteroides,* and *Bifidobacterium* genera was found in C57BL/6 pregnant mice, supporting the idea of gut microbiome changes during pregnancy (Gohir et al., 2015). These changes during pregnancy do not influence only the mother, but also impact the offspring’s neurodevelopment (Al Rubaye et al., 2021), e.g., altering the offspring’s microglial gene signature: male offspring of germ-free dams showed changes in the expression of 1216 microglial genes near birth. In contrast, the major impact on female offspring was observed in adulthood with alterations in 433 genes expressed by microglia compared to the offspring of dams with intact gut microbiota (Thion et al., 2018). Therefore, the maternal gut microbiota can also influence the offspring’s brain.

Changes in the microbiota are not limited to physiological situations such as pregnancy. In metabolic diseases such as obesity, there is evidence demonstrating changes in the bacterial profile, such as an increase in Firmicutes and a decrease in Bacteroidetes compared to their lean counterparts both in obese humans (Crovesy et al., 2020) and in HFD-fed animals (Jo et al., 2021). Indeed, the interaction between obesity and pregnancy causes specific changes that impact both mother and offspring’s microbiota. Mice exposed to HFD before mating and during gestation had increased *Akkermansia* (phylum Firmicutes) and *Bifidobacterium* compared to lean pregnant animals (Gohir et al., 2015). Previous studies show that the type of birth delivery can profoundly impact the bacterial profile in children (Ferretti et al., 2018; García-Mantrana et al., 2020), even though bacterial colonization does not occur in the uterus (Perez-Muñoz et al., 2017). The cohort study carried out by Chu et al. (2016) showed differences in the intestinal microbiota of children of mothers who consumed HFD compared to balanced diets, which demonstrated that the maternal diet influences both the mother and the infant’s bacterial profile.

Advances in the field have been showing how gut microbiota could influence the development of mood disorders such as anxiety and depression (Peirce and Alviña, 2019; Huang and Wu, 2021). In a recent study, pregnant women had their microbiota sequenced through stool samples during the third trimester of pregnancy, and children’s behaviour was analyzed at 2 years of age. Interestingly, increased alpha diversity, a measure of microbiota diversity, was related to less internalizing behaviour at 2 years of age, which can be associated with decreased anxiety behaviour. Furthermore, the butyrate-producing families *Lachnospiraceae* and *Ruminococcaceae* were found in mothers from children with normal behaviour. Prenatal exposure to healthy diets was also linked to the development of healthy behaviour in children (Dawson et al., 2021). However, few studies explore this mechanism in the context of maternal overnutrition and offspring’s behaviour.

Animal models support that maternal consumption of unhealthy diets during pregnancy, such as HFD, is related to gut microbiota changes that can predispose behaviours related to anxiety and depression in the offspring (Buffington et al., 2016; Bruce-Keller et al., 2017). To isolate the effects of obesity-modified microbiota on offspring behaviour, gut microbiota was transplanted from HFD-fed animals to female mice with depleted microbiota before copulation. It resulted in altered offspring’s microbiota and primed anxiety-like behaviour in males, but not in females. Male offspring from HFD transplanted dams also showed a pattern of depressive-like behaviour. Therefore, maternal microbiota influenced by the diet can lead to depressive and anxiety-like behaviours in the offspring in a sex-dependent manner (Bruce-Keller et al., 2017). In another preclinical study, co-housing the offspring of HFD dams with offspring of a control dam reversed changes in the offspring’s microbiota caused by maternal obesogenic diet and alleviated the behavioural abnormalities. Moreover, faecal transplantation from offspring of HFD dams to germ-free mice led to behavioural effects similar to those observed in the offspring of obese dams (Buffington et al., 2016).

**FIGURE 1:**
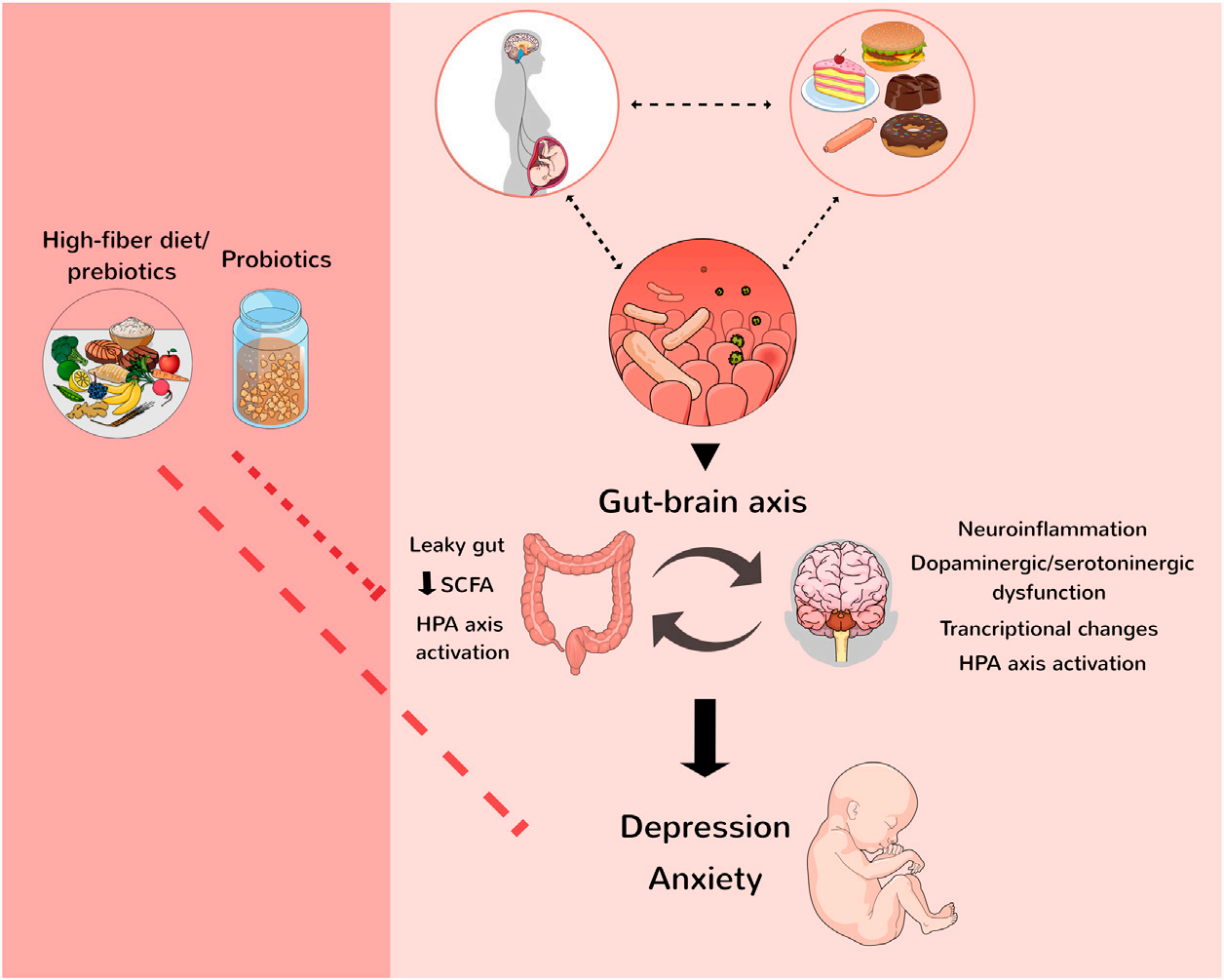
Maternal diet can affect the offspring, increasing the chances of metabolic and neurodevelopmental diseases. The gut-brain axis modulates anxiety and depression development since maternal microbiota influence the offspring’s behaviour. Some mechanisms showed in the figure are proposed to influence the development of neurological disorders such as psychiatric manifestations. The consumption of a high-fiber diet, prebiotics, and probiotics is helpful to modulate the offspring’s microbiome and, thus, can diminish the risk of mental illnesses. HPA, hypothalamus-pituitary-adrenal; SCFA, short-chain fatty acids. Figure created by the authors with the assistance from Mind the Graph (mindthegraph.com).

Among the proposed mechanisms, a decrease in fermenting bacteria responsible for SCFA production such as butyrate, propionate, and acetate can lead to a leaky gut, impairing the intestinal barrier and allowing immunogenic molecules from the gut, e.g., lipopolysaccharide (LPS) reach the circulation, where they activate inflammatory responses that can become chronic and lead to psychiatric manifestations (Dash et al., 2015; Winter et al., 2018). Yucatan pigs dams consuming CAF during pregnancy and lactation showed a decrease in SCFA in both dams’ and offspring’s faeces 100 days after birth. A decrease in neurogenesis markers in the offspring’s hippocampus was also found, evidencing the involvement of the gut-brain axis in this context (Val-Laillet et al., 2017). Also, altered microbiota during gestation can trigger maternal immune activation, which leads to conditions in the offspring that also have anxiety as a feature, e.g., autism spectrum disorder (Careaga et al., 2017; Gottfried and Bambini-Junior, 2018). Important to mention, gut microbiota changes impact the synthesis of neurotransmitters such as serotonin, dopamine, and norepinephrine [for review see (Huang and Wu, 2021)], which also might influence the development of mood disorders. Moreover, diet-induced maternal dysbiosis has also been proposed to increase hypothalamus-pituitary-adrenal (HPA) axis activation (Farzi et al., 2018; Di Gesù et al., 2021), whilst the activation of the HPA axis has an established relationship with psychiatric disorders such as anxiety (Frankiensztajn et al., 2020) (Figure 1).

Therefore, microbiota and its metabolites may provide an interesting mechanism of how the gut-brain axis is involved in the effects of obesity on the offspring’s brain and also provide a potential therapeutic target. The use of prebiotics and probiotics has provoked interest in the scientific community in recent years. While probiotics can modulate the composition of intestinal bacteria, prebiotics are the substrate used for fermentation and production of metabolites with an interest to improve host health. Recent research data indicate positive impacts of these strategies regarding metabolic diseases and also in the CNS, including improvement of cognitive impairment, anxiety, and depression [for a review see (Liu et al., 2019; Paiva et al., 2020)]. The gestation period could be a suitable and opportune moment to introduce, or even prioritize the probiotics therapy to benefit the offspring. In an animal model, consumption of probiotics concomitant with an HFD started before pregnancy and continued until lactation was able to protect mice offspring against metabolic disturbances compared to offspring of dams on HFD alone (Guo et al., 2018). On the other hand, its effect in pregnant women is controversial since promising literature (Desai et al., 2021) contrasts with evidence such as a randomized clinical trial that found no benefits with *Lactobacillus rhamnosus* and *Bifidobacterium lactis* supplementation in obese pregnant women regarding depression and well-being (Dawe et al., 2020). Therefore, future studies are needed to robustly assert whether probiotic supplementation during pregnancy would be useful to prevent the development of mood disorders such as depression and anxiety in the progeny.

## Current Research Gaps and Conclusion

As already mentioned, dysbiosis may be related to the genesis of chronic inflammation in obesity and, through the gut-brain axis, trigger neuroinflammation and the development of psychiatric manifestations (Peirce and Alviña, 2019; Milano et al., 2020; Shi et al., 2020). Maternal diet influences offspring’s brain development, e.g., affecting microglial maturation and gene expression (Thion et al., 2018) and also gut microbiota composition (Buffington et al., 2016). Moreover, brain cells such as microglia can be influenced by the gut-brain axis acquiring a more pro-inflammatory profile and triggering behavioural disturbances (Duan et al., 2021).

In this review, we highlighted the influence of maternal overnutrition and obesity on offspring’s CNS manifestations, mainly on depression and anxiety, psychiatric disorders increasingly prevalent in the world. We also show how the gut-brain axis is altered during pregnancy and obesity. Accordingly, healthier food consumption during pregnancy must be encouraged to prevent this global health issue (Hanson et al., 2017; Bhagavata Srinivasan et al., 2018).

Depression and anxiety may manifest at different stages of life, either in childhood, adulthood, or aging. Whenever it happens, the causes regarding maternal exposure cannot be excluded. Thus, a particularly productive focus for research would be to better understand the influence of the microbiota of obese mothers on the offspring’s brain, allowing the establishment of the importance of therapeutic interventions in this context. Modulating the microbiota through a healthier diet can be beneficial to the offspring, as maternal and offspring dietary fiber consumption was demonstrated to diminish cognitive and social disabilities while improving synaptic function and microglia maturation in the offspring (Liu et al., 2021). The use of prebiotics and probiotics has shown promising results to modulate the maternal and offspring microbiota, and it could be an interesting strategy to prevent psychiatric conditions, such as anxiety and depression (Sanders et al., 2019; Lee, 2021). However, conflicting results and the lack of knowledge about the mechanisms of action of these interventions should encourage future research focused on this subject.
